# Increased TGFβ1 and SMAD3 Contribute to Age-Related Aortic Valve Calcification

**DOI:** 10.3389/fcvm.2022.770065

**Published:** 2022-07-19

**Authors:** Mrinmay Chakrabarti, Aniket Bhattacharya, Mengistu G. Gebere, John Johnson, Zeeshan A. Ayub, Ioulia Chatzistamou, Narendra R. Vyavahare, Mohamad Azhar

**Affiliations:** ^1^Department of Cell Biology and Anatomy, School of Medicine, University of South Carolina, Columbia, SC, United States; ^2^Department of Neuroscience and Cell Biology, Child Health Institute of New Jersey Rutgers-Robert Wood Johnson Medical School, New Brunswick, NJ, United States; ^3^Department of Pathology, Microbiology, and Immunology, School of Medicine, University of South Carolina, Columbia, SC, United States; ^4^Biomedical Engineering, Clemson University, Clemson, SC, United States; ^5^William Jennings Bryan Dorn VA Medical Center, Columbia, SC, United States

**Keywords:** *Tgfb* = transforming growth factor beta, *Smad3*, Klotho, CAVD (calcific aortic valve disease), aortic valve calcification

## Abstract

**Aims:**

Calcific aortic valve disease (CAVD) is a progressive heart disease that is particularly prevalent in elderly patients. The current treatment of CAVD is surgical valve replacement, but this is not a permanent solution, and it is very challenging for elderly patients. Thus, a pharmacological intervention for CAVD may be beneficial. In this study, we intended to rescue aortic valve (AV) calcification through inhibition of TGFβ1 and SMAD3 signaling pathways.

**Methods and Results:**

The *klotho* gene, which was discovered as an aging-suppressor gene, has been observed to play a crucial role in AV calcification. The *klotho* knockout (*Kl*^–/–^) mice have shorter life span (8–12 weeks) and develop severe AV calcification. Here, we showed that increased TGFβ1 and TGFβ-dependent SMAD3 signaling were associated with AV calcification in *Kl*^–/–^ mice. Next, we generated *Tgfb1*- and *Smad3*-haploinsufficient *Kl*^–/–^ mice to determine the contribution of TGFβ1 and SMAD3 to the AV calcification in *Kl*^–/–^ mice. The histological and morphometric evaluation suggested a significant reduction of AV calcification in *Kl*^–/–^; *Tgfb1*^±^ mice compared to *Kl*^–/–^ mice. *Smad3* heterozygous deletion was observed to be more potent in reducing AV calcification in *Kl*^–/–^ mice compared to the *Kl*^–/–^; *Tgfb1*^±^ mice. We observed significant inhibition of *Tgfb1*, *Pai1*, *Bmp2, Alk2*, *Spp1*, and *Runx2* mRNA expression in *Kl*^–/–^; *Tgfb1*^±^ and *Kl*^–/–^; *Smad3*^±^ mice compared to *Kl*^–/–^ mice. Western blot analysis confirmed that the inhibition of TGFβ canonical and non-canonical signaling pathways were associated with the rescue of AV calcification of both *Kl*^–/–^; *Tgfb1*^±^ and *Kl*^–/–^; *Smad3*^±^ mice.

**Conclusion:**

Overall, inhibition of the TGFβ1-dependent SMAD3 signaling pathway significantly blocks the development of AV calcification in *Kl*^–/–^ mice. This information is useful in understanding the signaling mechanisms involved in CAVD.

## Introduction

Calcific aortic valve disease (CAVD) is a progressive heart disease in which aortic valve sclerosis progresses to aortic valve stenosis with severe calcification and impaired leaflet function (1–5). The aortic valve (AV) calcification affects 25% of the population over 65 years of age and about 50% of those over 85 years (2). Surgical valve replacement is the most effective treatment for valvular heart disease (6). Old age and chronic kidney disease (CKD) are important risk factors for CAVD (1). The serum levels of klotho (KL) decrease in CKD patients with heart valve calcification and therefore klotho is considered an independent risk factor for CAVD in CKD (7). The *klotho* gene (*Kl*) is identified as an anti-aging gene in mice that prolongs the life span (8). Since its serendipitous discovery, *klotho* has drawn significant attention owing to its potential role in aging, chronic kidney disease (CKD), and several cardiovascular diseases (9, 10). Earlier investigations indicated that *klotho* expresses predominantly in the distal tubular epithelial cells of the kidney (11, 12). Another study has confirmed klotho expression in human artery and cardiac myocytes (10). Functions of klotho include regulation of energy metabolism, anti-inflammatory and anti-oxidative effects, modulation of ion transport, and regulation of mineral metabolism (13). Several reports also endorsed the protective role of *klotho* in several organs as well as reversal of disease mechanisms including cardiovascular disease (14). The *Kl* encodes a single-pass transmembrane protein of 135 kDa. The klotho protein is clipped on the cell surface by membrane-anchored proteases and the entire extracellular domain (∼130 kDa) is released into the systemic circulation (8, 15, 16). Thus, klotho protein exists in two forms: membrane-bound klotho and secreted klotho (10, 17). More probably, membrane klotho solely serves as a co-receptor for the binding to fibroblast growth factor 23 (FGF23) (18). FGF23 is a bone-derived hormone that acts on kidney to stimulate phosphate elimination into urine and inhibit vitamin D synthesis, thereby modulating negative phosphate balance (19, 20). One significant characteristic of FGF23 is that it has very poor affinity to FGF receptors (21, 22). Binding of membrane klotho generates a constitutive binary complex with FGF receptors (FGFRs) which creates a *de novo* high-affinity binding site for FGF23 (22). Thus, FGF23 requires membrane klotho to bind to its cognate FGF receptors and exert its biological activity (22). The klotho (*Kl*^–/–^) and/or *Fgf23* (*Fgf23*^–/–^) knockout mice exhibit many key aspects associated with human CAVD including, premature aging, kidney disease, increased serum phosphate levels (i.e., hyperphosphatemia), and increased osteogenic gene expression (7, 23, 24). The *Kl*^–/–^ mice die by 12 weeks of age due to multiple age-related complications. The *Kl*^–/–^ mice develop calcific nodules in the AV hinge and aortic annulus, but they do not show significant AV leaflet thickening, extracellular matrix (ECM) disorganization, or inflammation (25). In addition, the *Kl*^–/–^ mice also develop ectopic calcification of aorta and kidneys (26). It has been reported that valve interstitial cells (VIC) in the calcified AV hinge in *Kl*^–/–^ mice become activated and express several pro-calcific markers, including COX2, RUNX2, osteopontin (OPN1/SPP1), and alkaline phosphatase (ALP). Recently, *Kl*^–/–^ mice have been successfully used in preclinical testing of potential new drugs for AV calcification (27). Since CKD is associated with klotho and human CAVD (28) and the AV calcification in *Kl*^–/–^ mice resembles human CAVD (29), the *Kl*^–/–^ mice are useful mouse models for investigating the mechanisms involved in pathogenesis of CAVD (29, 30).

Surgical specimens of the aortic valve (AV) obtained from older patients with CAVD have increased levels of transforming growth factor beta1 (TGFβ1) (31–37). Elevated TGFβ1 levels are frequently observed during vascular calcification and CKD (38–40) and contribute to the progression of calcification of cardiovascular tissues (41, 42). Both AV calcification and vascular calcification are regarded as the crucial risk factors in CKD and are associated with cardiovascular and all-cause morbidity and mortality (43) (44). The AV calcification is an active process with some parallels to physiological bone formation (3, 45). A critical role is attributed to vascular smooth muscle cells (VSMC) and VIC, which can differentiate and convert into myofibroblasts, osteoblast, and chondroblast-like phenotypes (46, 47). Osteoblasts or chondrogenic VSMCs/VICs actively promote valvular or vascular tissue mineralization (25, 48). Although the intracellular signaling pathways that control this trans-differentiation into myofibroblasts, osteoblasts, or chondrocytes are yet not understood completely (49). Human myxomatous valve disease (MMVD) is characterized by increased VIC proliferation, leaflet thickening, increased proteoglycan expression, and ECM remodeling (50). Cell proliferation is unaffected and αSMA expression is reduced in calcified AV of *Kl*^–/–^ mice (25), suggesting that VIC activation or MMVD is not present in *Kl*^–/–^ mice. TGFβ1 plays a crucial role in both myofibroblasts and osteogenic trans-differentiation of the VSMCs/VICs and induces cellular senescence through the upregulation of plasminogen activator inhibitor (*Pai1*) (38, 47). Moreover, TGFβ1-dependent osteoinductive signaling involves altered expression of the chondrogenic transcription factor SRY-Box 9 (SOX9), which is involved in AV calcification (51–54).

Increased levels of TGFβ ligands can contribute to cell degeneration, inflammation, metabolic malfunction, tissue fibrosis, and calcification. While the roles of TGFβ signaling depend on cellular contexts, age-related changes are also a potential context, and therefore the relationship between TGFβ signaling and klotho, which is involved in cellular senescence and aging-related diseases such as CAVD, warrants serious attention. There are many investigations that showed that *Kl*^–^*^/^*^–^ mice can induce calcification in aortic valves (25, 27, 55, 56). The AV of *Kl*^±^ mice do not show any calcification, although a high-fat diet resulted in collagen-I deposition and fibrosis of the aortic valve cusps on the aortic side (57). The signaling mechanisms of AV calcification in klotho deficient mice are still unclear and require further investigations. In this work, we investigated the impact of reduced TGFβ signaling on the AV calcification in *Kl*^–/–^ mice. We observed that partial inhibition of TGFβ signaling through haploinsufficiency of *Tgfb1* and *Smad3* improves the pathological condition of the AV calcification in klotho-deficient mice.

## Materials and Methods

### Ethics Statement

All animal procedures were performed according to the Guidelines for the Care and Use of Laboratory Animals published by the National Institutes of Health and were approved by the Institutional Animal Care and Use Committee (IACUC) of the University of South Carolina. Mice were euthanized by an overdose of isoflurane in a sealed container as approved by the IACUC.

### Mouse Strains

*Kl*^±^ (B6;129S5-*Kl*^TM 1L^*^ex^/Mmucd*, Stock# 011732) mice were obtained from the Mutant Mouse Resource and Research Centers (MMRRC) supported by the NIH. These mice were first backcrossed on to C57BL/6 background for more than nine generations. First, the *Kl*^±^*; Tgfb1*^±^ and *Kl*^±^*; Smad3*^±^ mice were generated in our mouse facility by the genetic crossing of the *Kl*^±^ (B6) to *Tgfb1*^±^ (50% 129SvJ and 50% CF-1) (58) and *Smad3*^±^ (129/SvJ) (59) mice. Self-crossing of *Kl*^±^*; Tgfb1*^±^ and *Kl*^±^; *Smad3*^±^ male and female mice resulted in the generation of wild-type control (*Kl*^+/+^, *Kl*^+/+^; *Tgfb1*^+/+^, and *Kl*^+/+^; *Smad3*^+/+^), *Kl*^–/–^, *Kl*^–/–^*; Tgfb1*^±^, and *Kl*^–/–^*; Smad3*^±^ mice. Before starting each study, we collected the tail at the age of 3 weeks and genomic DNAs were extracted for each animal and confirmed the genotype using gene-specific primers for *Kl*, *Tgfb1*, and *Smad3* (Table 1). PCR genotyping was done as described earlier (60–62).

**TABLE 1:** Primer sequence for PCR amplification.

Sl. No	Primer name	Sequence (5′–3′)	Amplified gene
1.	KL01	GCAGCGCATCGCCTTCTATC	*Klotho*
2.	KL02	ATGCTCCAGACATTCTCAGC	
3.	KL03	GATGGGGTCGACGTCA	
4.	KL04	TAAAGGAGGAAAGCCATTGTC	
5.	IMF36	AGGACCTGGGTTGGAAGTG	*Tgfb1*
6.	IMR36	CTTCTCCGTTTCTCTGTCACCCTAT	
7.	IMF11	GCCGAGAAAGTATCCATCAT	
8.	IMF-37	CGGCGAGGATCTCGTCGTGACCCA	*Smad3*
9.	IMR-37	GCGATACCGTAAAGCACGAGGAAG	
10.	IMF-38	GGATGGTCGGCTGCAGGTGTCC	
11.	IMR-38	TGTTGAAGGCAAACTCACAGAGC	

### Tissue Collection and Processing for Histology

Wild-type controls *Kl*^–/–^, *Kl*^–/–^*; Tgfb1*^±^, and *Kl*^–/–^*; Smad3*^±^ mice were sacrificed, and aortic valve tissues were collected for histological, immunohistochemical, morphometric, and molecular analyses. The whole heart of each mouse was perfused with 1XPBS and later fixed in 4% paraformaldehyde in PBS (Fisher Scientific, Waltham, MA, United States) for 48 h. Then, tissues were dehydrated in 70, 95, and 100% ethanol (Fisher Scientific, Waltham, MA, United States) and cleared with xylene (Fisher Scientific, Waltham, MA, United States). Paraffin-embedded hearts were sectioned using an RM2245 Leica microtome (Leica Biosystems, Buffalo Grove, IL, United States) and 7 μm thick serial sections through the aortic roots including annulus, aortic leaflets and hinge, and a portion of the sinus of Valsalva were collected for various histological and morphometric evaluation. Multiple serial sections from each animal were used for quantifying structural or histological changes using the NIH Image J (Fiji) software.

### Histology

All histological staining procedures were performed according to the protocol provided by the manufacturers. Before various histological staining procedures, the tissues were de-paraffinized and rehydrated. Hematoxylin and eosin staining (H&E staining) was carried out using Harris’ hematoxylin (Cat # HS-400) and Eosin (Sigma, Cat # HT110380). Tissue sections were kept in alizarin red stain (American MasterTech Scientific, Lodi, CA, United States) for 5 min for detection of calcium deposits. For alizarin red staining, tissue sections were fixed and dehydrated with –20°C cold acetone (Thermo Fisher Scientific, Waltham, MA, United States) and cleared with an acetone/xylene solution (50% acetone and 50% Safe Clear II Xylene substitute, both purchased from Thermo Fisher Scientific, Waltham, MA, United States). Alcian blue stain kit (catalog # KTABP2.5) was used to detect proteoglycans. The Verhoeff’s Elastin staining kit (Cat# KTVEL) was used for elastic fiber (black color) and collagens (red color) staining. Collagens were also detected by using the Manson’s Trichrome 2,000 stain kit (cat # KTMTR2PT). All kits for detecting proteoglycans, collagens, and elastin fibers were purchased from the American MasterTech Scientific (Lodi, CA, United States). At the end of each staining, the tissue slides were mounted with a permanent mounting medium (Vector Laboratories, Burlingame, CA, United States). All tissue sections were subsequently visualized and photographed in low and high magnifications under bright field optics on the Nikon Optiphot-2 (equipped with AxioCam MRc Camera) and EVOS TM FL Auto Imaging System (Thermo Fisher Scientific, Inc., Grand Island, NY, United States). Morphometric quantification of signal intensity was done on multiple serial sections from each animal per group by using the NIH- Image J (Fiji) software.

### Immunohistochemistry

Paraformaldehyde-fixed paraffin-embedded serial sections representing annulus, aortic leaflets and hinge, and a portion of the sinus of Valsalva were used for immunohistochemistry. Sections were de-paraffined and hydrated with two changes in 1X PBS and one change in deionized water (5 min each). Heat-mediated antigen retrieval was performed by dipping the slides in a mildly boiling 1X citric acid buffer (catalog no. S1700; Agilent Dako, Santa Clara, CA, United States) for 10 min in a microwave, cooled to room temperature, and rinsed in PBS. Endogenous peroxidases were blocked with freshly prepared 0.5% H_2_O_2_/methanol for 30 min, followed by non-specific epitope blocking with 5% goat serum/0.1% Tween/0.02% sodium azide in PBS for 20 min. Avidin and Biotin blocking was done as per the manufacturer’s recommendation (Cat# SP-2001, Vector Labs, Burlingame, CA, United States), followed by overnight incubation at 4°C in anti-pSMAD2 (1:3000, Millipore, Burlington, MA, United States) (61). Slides were then washed and incubated with appropriate biotinylated secondary antibody (1:200) for 30 min, followed by Avidin-Biotin complex (Cat# PK-6100, Vectastain Elite ABC HRP kit) for 30 min, washed in PBS, and finally developed with DAB/H_2_O_2_. Nuclei were counterstained with hematoxylin and sections were dehydrated through graded ethanol series, cleared in xylene, and mounted. Both low- and high-magnification images were taken under bright field optics on the Nikon Optiphot-2 (equipped with AxioCam MRc Camera). Morphometric quantification of pSMAD2-stained average area (μm^2^) was done on multiple serial sections from each animal per group by using the NIH- Image J software.

### RNA Isolation, cDNA Synthesis, and Quantitative PCR

The AV tissue was dissected manually from wild-type, *Kl*^–/–^, *Kl*^–/–^*; Tgfb1*^±^, and *Kl*^–/–^*; Smad3*^±^ mice. The AV tissue contained aortic roots including annulus, aortic leaflets and hinge, and a portion of the sinus of Valsalva. Five individual biological samples from each group were analyzed for gene expression study. Total RNA was isolated from the AV tissue of wild-type and *Kl*^–/–^, *Kl*^–/–^*; Tgfb1*^±^, and *Kl*^–/–^*; Smad3*^±^ mice using Trizol (Invitrogen/ThermoFisher, Grand Island, NY, United States) and miRNeasy micro kit (Qiagen, Germantown, MD, United States) according to the manufacturer’s protocols. cDNA was generated from 500 ng total RNA using an RT-PCR kit according to the instructions provided by the manufacturer (Bio-Rad Laboratories, Inc.). cDNA was then diluted 10 times and later subjected to quantitative PCR amplification (Bio-Rad CFX) using pre-validated gene-specific primers procured from the vendor (Bio-Rad Laboratories, Inc., Hercules, CA, United States) (Table 2). Approximately, 10 ng of cDNA was used for each 20 μl qPCR reaction. Following qPCR analyses, the cycle count threshold (Ct) was normalized to species-specific housekeeping genes (*B2m*; purchased from Bio-Rad, Inc.) and the 2^^–Δ^*^Ct^* values were determined and graphically presented. Statistically significant differences in gene expression levels were determined using Student’s *t*-test, indicated in the figure legends, on at least three or more independent experiments with *p* < 0.05 considered significant.

**TABLE 2:** List of qPCR primers.

Sl. No	Target gene	Biorad unique assay ID
1.	Tgfb1, mouse	qMmuCED0044726
2.	Tgfbr2, mouse	qMmuCID0015359
3.	Alk2, mouse	qMmuCID0040095
4.	Pai1, mouse	qMmuCID0027303
5.	Bmp2, mouse	qMmuCID0014251
6.	SPP1, mouse	qMmuCED0040763
7.	Runx2, mouse	qMmuCID0005205
8.	B2m, mouse	qMmuCID0040553

### Western Blot Analysis

Three independent “pooled” samples of the AV tissue from each group of mice were used for western blotting. The AV tissues in each sample were “pooled” from two individual mice for each genotype (wild-type, *Kl*^–/–^, *Kl*^–/–^*; Tgfb1*^±^, and *Kl*^–/–^*; Smad3*^±^). Thus, each independent pooled sample consists of two biological replicates (*n* = 6 per genotype). The AV tissue contained aortic roots including annulus, aortic leaflets and hinge, and a portion of the sinus of Valsalva. The AV tissue was cut into small pieces and homogenized using Wheaton tapered tissue grinders (Thermo Fisher Scientific, Rockford, IL, United States) in M-PER mammalian protein extraction reagent (Thermo Fisher Scientific) with a complete mini protease inhibitor cocktail (Sigma-Aldrich, St. Louis, Louis, MO, United States) and Halt protease and phosphatase inhibitor single-use cocktail (Thermo Fisher Scientific, Rockford, IL, United States) as per the manufacturer’s protocol. Homogenized tissue lysates were subjected to brief sonication for 20 s on ice and then kept at room temperature for 20 min. Then, centrifugation was performed at 15,000 rpm for 20 min at 4°C and the supernatants were collected. Total protein concentration in the supernatant was determined using the Pierce BCA protein assay kit (Thermo Scientific, Rockford, IL, United States). Samples were stored at –80°C until further use. Western blotting was performed with equal amounts of protein samples and the primary IgG antibodies against phospho-SMAD2 (Cell Signaling Technology, Danvers, MA, United States Cat #3108), SMAD2 (Cell Signaling, Cat #5339), phospho-SMAD3 (Cell Signaling, Cat #9520), SMAD3 (Cell Signaling, Cat #9523), phospho-SMAD1/5 (Cell Signaling, Cat #9516), SMAD1/5 (Cell Signaling, Cat #9743), phospho-p38 (Cell Signaling, Cat #4511), p38 (Cell Signaling, Cat #8690), phospho-ERK1/2 (Cell Signaling, Cat #4370), and ERK1/2 (Cell Signaling, Cat #4695) at a dilution of 1:1000. Primary IgG antibodies against all these proteins were purchased from Cell Signaling Technology, Inc. (Danvers, MA, United States). The horseradish peroxidase-conjugated anti-mouse or anti-rabbit secondary IgG antibody (Cell Signaling, Cat # 7074) was used at 1:5000 dilution to detect a primary IgG antibody. Western blots were incubated with Clarity western ECL detection reagents (Bio-Rad Laboratories, United States) and exposed to X-OMAT AR films (Eastman Kodak, Rochester, NY, United States) for autoradiography. The autoradiograms were scanned on an EPSON Scanner using Photoshop software (Adobe Systems, Seattle, WA, United States). β-actin, clone AC-15, monoclonal primary antibody (Sigma-Aldrich, St. Louis, MO, United States) was used as a loading control to compare equal loading in the SDS-PAGE. We quantified our results *via* densitometric analysis using NIH Image J (Fiji) software. Quantitative densitometric analysis normalized the expression levels of phosphorylated proteins with their non-phosphorylated/total forms or β-actin. Microsoft Excel was used for recording and managing the raw data. Statistics were performed using pair-wise comparisons between the groups, utilizing analysis of variance and unpaired two-tailed Student’s *t*-test (GraphPad Prism 9 Software, San Diego, CA). Data were reported as means ± *SD* of the mean. Probability values < 0.05 were considered significant.

## Results

### Increased *Tgfb1* Expression and SMAD3 Activation Are Associated With the Calcification of the Aortic Valve in Klotho Knockout Mice

**FIGURE 1:**
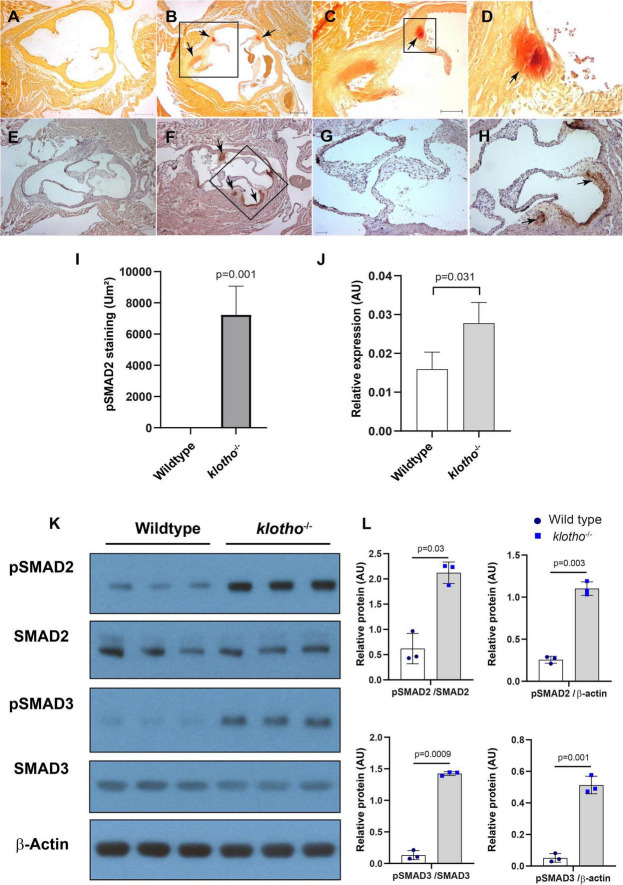
The klotho (*Kl*) genetic deletion leads to calcification in the aortic valve hinge and annulus with upregulation of *Tgfb1* and TGFβ-dependent SMAD2 signaling in 10–12-week-old mice. **(A–D)** Alizarin red staining of wild-type **(A)** and *Kl*^–/–^ **(B–D)** mice. Scale bars = **(A–B)** 200 μm; **(C)** 100 μm; **(D)** 50 μm. **(E–I)** Immunohistochemistry showing localized pSMAD2 levels in AV hinge and aortic annulus of wild-type **(E,G)** and *Kl*^–/–^ **(F,H)**. The pSMAD2-stained area was quantified using NIH Image J software **(I)**. **(J)** qPCR study to quantify *Tgfb1* expression in the pooled and micro-dissected tissue samples of AV and annulus from wild-type and *Kl*^–/–^ mice. **(K)** Western blotting analyses showing levels of phosphorylated SMADs (p SMAD2, pSMAD3), total SMADs (SMAD2, SMAD3), and β-actin in micro-dissected and pooled tissue samples containing AV and annulus from wild-type and *Kl*^–/–^ mice. **(L)** Densitometric analysis quantifying band intensities of western blots. The densitometry graphs of pSMAD2 and pSMAD3 were normalized with total or non-phosphorylated form of the proteins or β-actin. Values indicate mean ± SD, and significant “*p*-values” between wild-type and *Kl*^–/–^ groups were given on the top of histograms.

We collected tissues from the aortic valve from 8- to 10-week-old wild-type and *Kl*^–/–^ mice and performed alizarin red staining of tissue sections to determine calcification in the aortic valve area. Our alizarin red staining confirmed the presence of calcific nodules in the AV hinge and aortic annulus of the *Kl*^–/–^ mice (*n* = 12) (Figures 1A–D). There was no AV calcification seen in the control mice, which included *Kl*^+/+^ and *Kl*^±^ mice. Immunohistochemistry analysis indicated that increased phosphorylated SMAD2 (pSMAD2) was associated with AV calcification in the hinge and aortic annulus (*n* = 6) (Figures 1E–I). We tested if the *Tgfb1* mRNA level was upregulated in the AV tissue of *Kl*^–^*^/^*^–^ mice by qPCR analysis. The data indicated significant upregulation of *Tgfb1* mRNA expression (*n* = 5, *p* = *0.031*) in *Kl*^–/–^ mice compared to the wild-type control (Figure 1J). Next, we determined if the increased *Tgfb1* mRNA expression level is consistent with the induction of the downstream TGFβ signaling molecules at the protein level. We analyzed the downstream TGFβ signaling pathway molecules in tissue samples collected from the AV tissue of wild-type and *Kl*^–^*^/^*^–^ mice. Since phosphorylation of SMAD2 and SMAD3 is typically used as a surrogate for TGFβ signaling, western blot analysis was used to quantify both phosphorylated and total (no-phosphorylated) SMAD and SMAD3. The data showed statistically significant induction of activated forms of the SMAD2 (i.e., pSMAD2) and SMAD3 (i.e., pSMAD3) (TGFβ-specific SMADs), in *Kl*^–/–^ mice when compared to wild-type mice (*n* = 6) (Figures 1K,L).

### Genetic Inactivation of TGFβ Signaling Pathway Components Improved Structural Features and Extracellular Matrix Organization of Aortic Valve in Klotho-Deficient Mice

**FIGURE 2:**
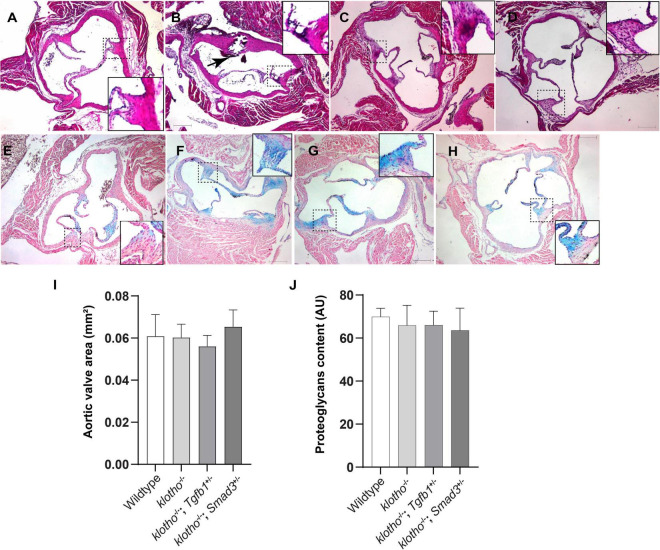
Effect of lowering of TGFβ1 and SMAD3 on AV structure and proteoglycan (GAG) distribution in 10- to 12-week-old *Kl*^–/–^ mice. **(A–D)** Hematoxylin and eosin (H&E) staining and **(E–H)** Alcian blue staining of wild-type, *Kl*^–^*^/^*^–^, *Kl*^–^*^/^*^–^*;Tgfb1*^±^, and *Kl*^–^*^/^*^–^*;Smad3*^±^ mice showing aortic valve histology and proteoglycans distribution. A magnified view showing further details of the boxed region is presented for each image. **(I–J)** Quantitative analysis of AV area in H&E-stained **(I)** and proteoglycans content in alcian blue-stained **(J)** images. Quantification of AV area and GAG content was done on multiple serial sections from each animal per group by using NIH- Image J software. Values indicate mean ± *SD* (*n* = 5). Scale bars = **(A–H)** 200 μm.

**FIGURE 3:**
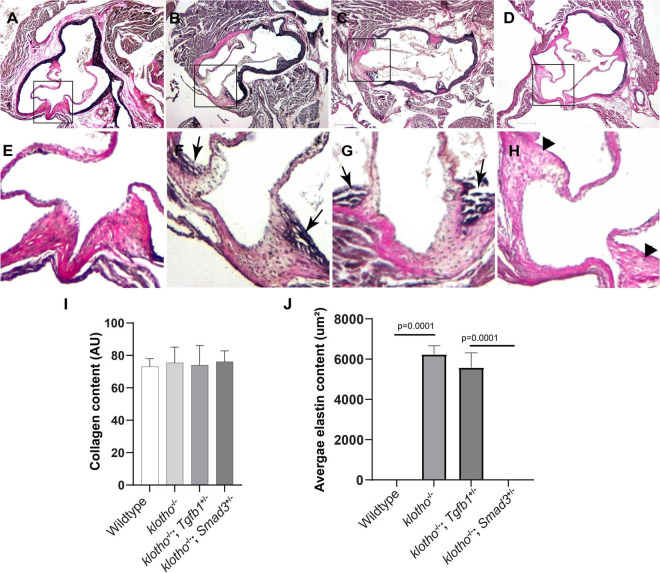
Effect of *Tgfb1* and *Smad3* heterozygous deletion on collagen and elastin fibers in the aortic valve of 10–12-week-old *Kl*^–/–^ mice. **(A–H)** Verhoeff-Van Gieson (VVG) staining of tissue sections through the aortic valve and annulus of wild-type **(A,E)**, *Kl*^–^*^/^*^–^ **(B,F)**, *Kl*^–^*^/^*^–^*;Tgfb1*^±^ **(C,G)**, and *Kl*^–^*^/^*^–^*;Smad3*^±^ **(D,H)** mice. Magnified images of the boxed area **(A–D)** are given in **(E–H)**. Arrows indicate the presence of abnormal elastin fibers in *Kl*^–/–^ **(F)** and *Kl*^–/–^;*Tgfb1*^±^ **(G)** mice. VVG stains elastin in black and collagen fibers in red color. **(I)** Histogram showing total collagen content in the aortic valve, including leaflets, annulus, and AV hinge. Quantification of collagen fibers content was done by measuring the intensity of collagen staining on multiple serial sections from each animal per group by using NIH-Image J software. Values indicate mean ± *SD* (*n* = 5). **(J)** Histogram showing the average area containing the elastin fibers in the AV hinge. Quantification of elastin fibers content was done by measuring the average area in the AV hinge with elastin staining on multiple serial sections from each animal per group by using NIH-Image J software. Values indicate mean ± *SD* (*n* = 5), and significant “*p*-values” were given on the top of histograms. Scale bars: **(A–H)** 200 μm.

Histological staining was carried out to examine the morphological, cellular, and structural changes in the AV tissue of 8–10-week-old *Kl*^–/–^ mice following the partial genetic deletion of TGFβ1 and SMAD3 genes (Figures 2A–H). Histological examination of H&E-stained serial tissue sections confirmed calcified nodules in the AV hinge and annulus in the *Kl*^–/–^ mice (*n* = 12) (Figures 2A,B). Morphometric quantitative analysis indicated no significant changes in the overall thickening of AV leaflets in *Kl*^–/–^ mice compared to wild-type mice (*n* = 6) (Figure 2I). Histological and morphometric evaluation of *Kl*^–/–^*;Tgfb1*^±^ mice showed partial improvement in the overall tissue structure of the AV hinge and annulus compared to *Kl*^–/–^ mice (*n* = 6) (Figures 2A–C,I). Similar analysis of *Kl*^–/–^*;Smad3*^±^ mice revealed a complete rescue of the AV hinge and aortic annulus (Figures 2A–D,I). Alcian blue staining was used to observe chondrogenic differentiation and proteoglycans or glycosaminoglycans (GAGs) distribution in the AV hinge and aortic annulus in all four groups of mice (*n* = 6–12). The data indicated abnormal proteoglycans in the AV hinge and aortic annulus in both *Kl*^–/–^ (*n* = 12) and *Kl*^–/–^*;Tgfb1*^±^ (*n* = 6) mice compared to wild-type or *Kl*^–/–^*;Smad3*^±^ mice (*n* = 12), but morphometric quantification did not reveal any significant changes in the overall proteoglycans content (*n* = 6) (Figures 2E–H,J). Finally, we determined collagen and elastic fibers’ organization through the VVG staining of serial sections (*n* = 6–12) (Figures 3A–J). The quantitative analysis did not reveal any significant changes in overall collagen content (Figure 3I). Histological assessment of high-powered images and morphometric evaluation showed dysregulated elastic fiber organization in the AV hinge of *Kl*^–/–^ mice (*n* = 12), which was significantly rescued by partial genetic deletion of *Smad3* (*n* = 12) but not *Tgfb1* (*n* = 6) (Figures 3A–H,J).

### Haploinsufficiency of *Tgfb1* and *Smad3* Significantly Reduced Vascular Calcification in Klotho-Deficient Mice

**FIGURE 4:**
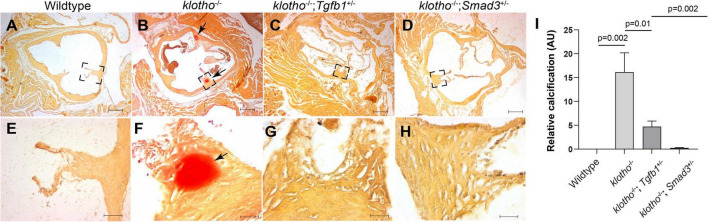
Partial genetic inactivation of *Tgfb1* and *Smad3* inhibits AV calcification in 10–12-week-old *Kl*^–/–^ mice. **(A–H)** Alizarin red staining of aortic valve in wild-type **(A,E)**, *Kl*^–^*^/^*^–^ **(B,F)**, *Kl*^–^*^/^*^–^*;Tgfb1*^±^ **(C,G)**, and *Kl*^–^*^/^*^–^*;Smad3*^±^ **(D,H)** mice. Images **(E–H)** are the magnified views of the corresponding boxed region **(A–D)**. Scale bars = **(A–D)**, 200 μm; **(E–F)**, 50 μm. **(I)** Quantitative analysis of calcification. Quantification of calcification was done on multiple serial sections from each animal per group by using NIH-Image J software. Values indicate mean ± *SD*, and significant “*p*-values” were given on the top of histograms.

We generated *Kl*^–/–^;*Tgfb1*^±^ and *Kl*^–/–^*;Smad3*^±^ mice to confirm the effect of TGFβ1 and canonical SMAD3-dependent TGFβ signaling pathways on AV calcification in klotho-deficient mice. Histological and quantitative analyses of alizarin red-stained serial sections through aortic roots including annulus, aortic leaflets and hinge, and a portion of the sinus of Valsalva of the 8- to 10-week-old wild-type, *Kl*^–/–^, *Kl*^–/–^;*Tgfb1*^±^ and *Kl*^–/–^*;Smad3*^±^ were performed (*n* = 6–12) (Figures 4A–I). There was no AV calcification in wild-type mice (Figures 4A,E). The *Kl*^–/–^ mice showed calcific nodules in the AV hinge (*n* = 12, *p* = *0.002*) (Figures 4A,B,E,F,I). Interestingly, the overall extent of the calcification in the AV hinge was less in *Kl*^–/–^*;Tgfb1*^±^ mice compared to the *Kl*^–/–^ mice (*n* = 6, *p* = *0.01*) (Figures 4A–C,E–G,I). Importantly, the AV calcification was almost completely rescued in *Kl*^–/–^*;Smad3*^±^ mice (*n* = 12, *p* = *0.002*) (Figures 4A–I).

### Heterozygous Deletion of *Tgfb1* and *Smad3* Blocks the Expression of Genes Involved in Aortic Valve Calcification

**FIGURE 5:**
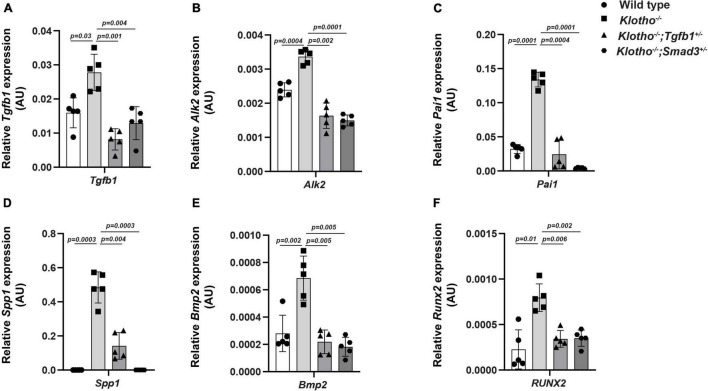
Effect of partial genetic deletion of *Tgfb1* and *Smad3* on key genes involved in TGFβ and BMP signaling, and AV calcification. The qPCR analysis of selected genes in micro-dissected pooled tissue samples representing AV leaflets and hinge and aortic annulus of wild-type, *Kl*^–^*^/^*^–^, *Kl*^–^*^/^*^–^*;Tgfb1*^±^, and *Kl*^–^*^/^*^–^*;Smad3*^±^ mice. Expression levels of *Tgfb1* (TGFβ ligand) **(A)**, *Alk2* (BMP Type I receptor) **(B)**, *Pai1* (TGFβ target gene) **(C)**, *Spp1* (calcification marker) **(D)**, *Bmp2* (BMP ligand) **(E)**, and *Runx2* (osteoblast differentiation marker) **(F)** were quantified. Triplicate samples from each of the five different biological replicates (*n* = 5) were analyzed. Quantitative data are shown as mean ± *SD* (*n* = 5). Two-sided Student’s *t*-test (unpaired) was used. Significant comparisons are indicated on the top of each histogram.

We have investigated the impact of partial genetic deletion of *Tgfb1* and *Smad3* on the expression of genes involved in TGFβ (*Tgfb1, Pai1*) and BMP (*Bmp2, Alk2*) signaling and osteoblast differentiation (*Spp1, Runx2*) during CAVD development and progression in *Kl*^–/–^ mice. The qPCR analysis was performed on aortic valve tissue containing aortic roots including annulus, sinus of Valsalva, and aortic leaflets and hinge from individual 8- to 10-week-old wild-type, *Kl*^–/–^, *Kl*^–/–^*;Tgfb1*^±^, and *Kl*^–/–^*;Smad3*^±^ mice (*n* = 5) (Figure 5). We observed significant increase in the *Tgfb1* (*p* = *0.03)*, *Pai1* (*p* = *0.0001*), *Bmp2* (*p* = *0.002*), *Alk2* (*p* = *0.0004*), *Spp1* (*p* = *0.0003*), and *Runx2* (*p* = *0.01*) mRNA expression in the AV tissue from the *Kl*^–/–^ mice compared to wild-type mice (Figure 5). Partial genetic inhibition of *Tgfb1* and *Smad3* significantly inhibited the expression of all these genes. Heterozygous *Smad3* deletion was observed to be more potent in reducing the expression of *Pai1* and *Spp1* genes in *Kl*^–/–^ mice compared to partial *Tgfb1* ligand inhibition (Figures 5C,D).

### Partial Genetic Deletion of *Tgfb1* and *Smad3* Blocks Aortic Valve Calcification by Reducing Both Canonical and Non-canonical TGFβ Signaling Pathways

**FIGURE 6:**
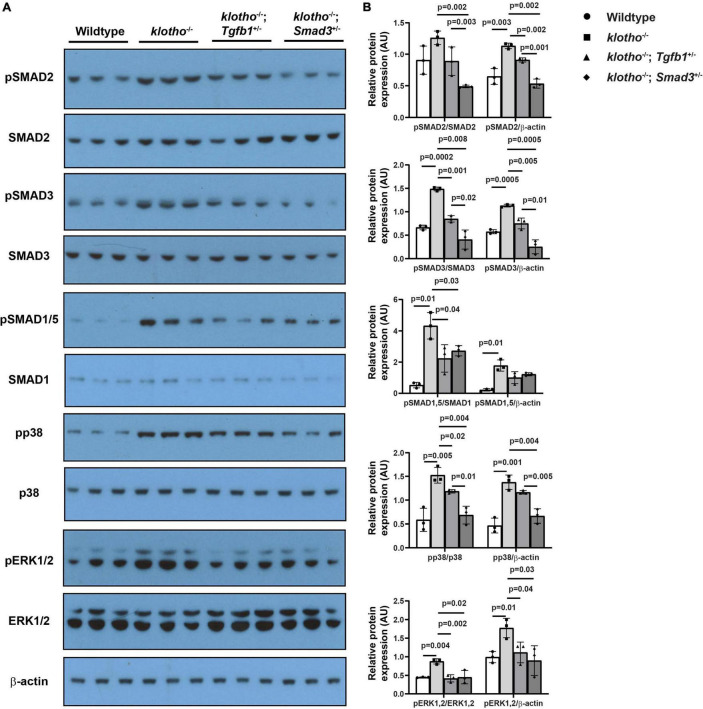
Haploinsufficiency of *Tgfb1* and *Smad3* inhibits canonical and non-canonical TGFβ signaling in the aortic valve of *Kl*^–/–^ mice. **(A)** Western blot analysis on three different micro-dissected pooled tissues, from two to three hearts/sample, representing AV leaflets and hinge area and aortic annulus of 10–12-week-old wild-type, *Kl*^–^*^/^*^–^, *Kl*^–^*^/^*^–^*;Tgfb1*^±^, and *Kl*^–^*^/^*^–^*;Smad3*^±^ mice showing protein levels of the phosphorylated forms of canonical and non-canonical pathway molecules (pSMAD2, pSMAD3, pSMAD1/5, pp38, and pERK1/2) and their non-phosphorylated/total forms (SMAD2, SMAD3, SMAD1/5, p38, and ERK1/2). A common and independent β-actin blot (bottom) was also used for normalizing the data. **(B)** Densitometric quantification of phosphorylated proteins after normalization to total non-phosphorylated proteins or β-actin. Numerical data (mean ± *SD*) from three pooled samples are presented as scatter plots with bar. The *p*-values are shown on top of the histograms.

We observed significant induction of TGFβ receptor-dependent phosphorylation of serine/threonine residues of TGFβ SMADs (i.e., pSMAD2 and pSMAD3), TGFβ/BMP SMAD1/5 (i.e., pSMAD1/5), and non-SMAD pathways (i.e., p38 MAPK, ERK1/2 MAPK) in the AV tissue from the *Kl*^–/–^ mice (*n* = 6) (Figure 6). Our immunoblot analyses of AV tissues from the wild-type control, *Kl*^–/–^, *Kl*^–/–^*;Tgfb1*^±^, and *Kl*^–/–^*;Smad3*^±^ mice indicated that haploinsufficiency of *Tgfb1* and *Smad3* reduced the activation of these molecules in the *Kl*^–/–^ mice (Figure 6A). There was no significant change in the total amount of the SMAD2, SMAD3, SMAD1/5, p38, ERK1/2 (ERK1 (top band), 44 KDa; ERK2 (bottom band), 42 KDa), and β-actin proteins in the wild-type control, *Kl*^–/–^, *Kl*^–/–^*;Tgfb1*^±^, and *Kl*^–/–^*;Smad3*^±^ mice (Figure 6A). Quantitative densitometric analysis comparing the expression levels of phosphorylated proteins with their non-phosphorylated/total forms or β-actin revealed similar results (Figure 6B). Collectively, partial *Smad3* deletion was more potent than heterozygous deletion of *Tgfb1* in reducing the activation of SMAD2/3/5, p38, and ERK1/2 MAPK during aortic valve calcification in klotho-deficient mice (Figures 6A,B).

## Discussion

The klotho deficiency has been shown to cause age-related calcification of AV hinge and aortic annulus in mice (25). The *Kl*^–/–^ mice also exhibit increased expression of many key osteogenic genes involved in human CAVD (25, 63). We demonstrated that upregulation of TGFβ1 and SMAD3 are involved in AV calcification in klotho-deficient mice. The data also indicate that calcific nodule formation in the aortic valve of klotho-deficient mice is associated with increased *Tgfb1* expression and elevated levels of activated forms of SMAD2, SMAD3, SMAD1/5, p38, and ERK1/2 MAPK.

Importantly, partial genetic ablation of *Tgfb1* or *Smad3* significantly decreases the expression of *Tgfb1* and both canonical (SMAD-dependent) and non-canonical (MAPK-mediated) TGFβ signaling pathways and blocks the pathological progression of the AV calcification in *Kl*^–/–^ mice. Collectively, while *Tgfb1* haploinsufficiency significantly improved the structural and ECM features, *Smad3* haploinsufficiency almost fully reversed pathological structural changes and disrupted elastin fiber organization in the aortic valve of klotho-deficient mice. Dysregulated elastin fibers in the AV leaflet hinge in *Kl*^–/–^ mice are consistent with a recent report by Gomez-Stallons et al. (64) showing increased elastin fragmentation and loss of elastin integrity in postmortem leaflet patients with CAVD.

Haploinsufficiency of *Smad3* has been observed to be more potent in inhibiting aortic valve calcification compared to *Tgfb1* ligand inhibition. This is consistent the notion that signaling from TGFβ1 and/or other TGFβ ligands could be effectively blocked by partial deletion of *Smad3*. Thus, the effect of lowering TGFβ1 on SMAD2/3 activation and AV calcification rescue may be somewhat less pronounced in *Kl*^–/–^*;Tgfb1*^±^ mice as compared to *Kl*^–/–^*;Smad3*^±^ mice. This may be due to functional compensation of the loss of TGFβ1 by other TGFβ ligands (i.e., TGFβ2, 3) and SMAD2. All three TGFβ ligands (TGFβ1, TGFβ2, and TGFβ3) interact with TGFβ Type I and II receptors and lead to activation of SMAD3 and SMAD2. Activated SMAD2/3 with SMAD4 translocate to the nucleus and regulate TGFβ target genes (e.g., *Pai1*) (36, 65). Increased levels of TGFβ1 (31) and pSMAD2 and PAI1 (66) are associated with CAVD. PAI1 is a known TGFβ target, and therefore, its expression was reduced along with the AV calcification rescue in response to partial inactivation of *Tgfb1* (*Kl*^–/–^*;Tgfb1*^±^ mice) or *Smad3* (*Kl*^–/–^*;Smad3*^±^ mice) in klotho-deficient mice. Wirrig et al. have reported increased expression of *Spp1* and *Runx2* in AV tissue of the *Kl*^–/–^ mice (55). Gomez-Stallons et al. (56) (ATVB) have reported that *Spp1, Bmp2*, and *Runx2* are increased in *Kl*^–/–^ AV tissue (56). Our results indicating significant rescue of CAVD and expression of these genes in *Kl*^–/–^ mice by partial genetic deletion of *Tgfb1* or *Smad3* are consistent with these published findings. It remains unclear if SMAD2 and SMAD3 play a redundant or unique role in AV calcification in CAVD.

There are several experimental limitations in this study. The effect of sex is not specifically addressed. The levels of active and latent TGFβ1 protein are not determined. Since most widely used anti-TGFβ1 antibodies (2G7, 1D11) cross-react to TGFβ3 and not TGFβ2 ligands (67), this study only demonstrated upregulation of *Tgfb1* transcript levels in the aortic valve of *Kl*^–/–^ mice. Regardless of these limitations, the molecular data indicate that both TGFβ1 and SMAD3 contribute to the expression of critical genes involved in the pathogenesis of CAVD. Collectively, our findings represent novel genetic approaches for blocking the progression of CAVD.

**FIGURE 7:**
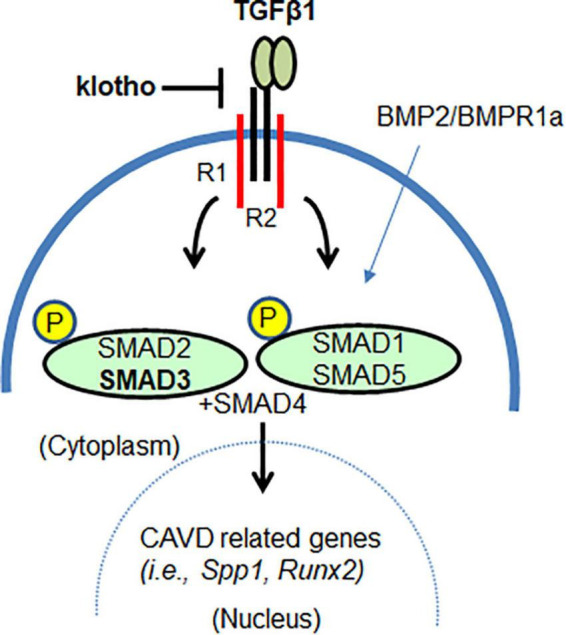
The klotho as a mediator of TGFβ1-dependent SMAD3 signaling in AV calcification. SMAD3 can mediate klotho-dependent TGFβ1 signaling. Lowering TGFβ1-dependent SMAD3 signaling also lowers SMAD1/5 signaling. Thus, klotho functions, in part, as a mediator by restraining or attenuating the augmented TGFβ/BMP signaling and TGFβ-regulated transcription of genes involved in CAVD.

Another important piece of information that emerged from this study is the effect of lowering TGFβ1 or SMAD3 on BMP signaling pathway in CAVD. TGFβs and the BMPs are members of the TGFβ family (68). It is well established that increased BMP signaling contributes to AV calcification in *Kl*^–/–^ mice. It has been reported that both *Bmp2* and pSMAD1/5 levels are increased in AV tissue of *Kl*^–/–^ mice and that genetic inactivation of BMP receptor Bmpr1a in the aortic valve interstitial cells can prevent AV calcification (56). It is remarkable to find that lowering of TGFβ1 as well as SMAD3 has significantly reduced the expression of BMP ligand (*Bmp2*) and BMP Type I receptor (*Alk2*) and activation of BMP-dependent SMAD (pSMAD1/5). Constitutively active mutants of ALK2 have been identified as causative of Fibrodysplasia Ossificans Progressiva (FOP), which is an extremely rare heritable disorder of connective tissues characterized by progressive heterotopic ossification in various skeletal sites (69). It is known that TGFβ1 *via* TGFβ Type I receptor can interact with ALK2 resulting in the simultaneous activation of SMAD2/3 and SMAD1/5 (70, 71). Also, *Alk2* deletion can reduce both SMAD2/3 and SMAD1/5 in cushion mesenchymal cells during heart development (72). Importantly, lowering pSMAD3 (this study) or pSMAD1/5 (56) has a similar effect in blocking AV calcification in *Kl*^–/–^ mice, suggesting crosstalk between TGFβ and BMP pathways in maintaining AV homeostasis (Figure 7). It is noteworthy that SMAD7, an inhibitory SMAD and TGFβ target gene, binds to and blocks all type I receptors in the TGFβ family, whereas SMAD6 shows preferential binding and inhibitory activity toward the BMP type I receptors ALK3 and ALK6 (73). Both SMAD 6 and 7 cooperate in suppressing the physiological BMP signaling during the differentiation of mesenchymal progenitor cells to osteoblasts (74). This is due to rapid and direct induction of *Smad6* expression by the primary BMP stimulus, followed by activation of autocrine TGFβ signaling, which then induces a second wave of *Smad7* expression that, together with the preexisting SMAD6, shuts down BMP receptor activity in a more sustained manner and limits the rate of differentiation to osteoblasts (74). Whether the negative regulation of TGFβ and BMP pathway *via* SMAD7 is failed in *Kl*^–/–^ mice remains a potential area of future investigation.

This study indicates that the haploinsufficiency of *Smad3* is more effective than *Tgfb1* heterozygous deletion in decreasing the activation of p38 MAPK in AV tissue of klotho-deficient mice. Improper activation of p38 or ERK MAPKs is a precursor of constitutive SMAD2/3 signaling associated with aortopathy (75, 76). TGFβ-activation of the SMAD2/3 requires the kinase activity of the TGFβRI, while the TGFβRI kinase activity is not required for the TGFβ-induced oligomerization of the TGFβRII/TGFβRI complex, where the TRAF6 binding to the TGFβRI results in the activation of TAK1-p38 MAPK pathway. Thus, TGFβ activation of TGFβRII/TGFβRI-complex leads to (a) the TGFβRI-kinase-dependent activation of SMAD2/3 and (b) the TGFβRII/TGFβRI-hetero-oligomerization which will cause a rapid activation of TRAF6 resulting in p38 MAPK pathway activation. It is possible that changes in the non-canonical TGFβ pathway may be a primary determinant of AV calcification in *Kl*^–/–^ mice. Thus, the exact contribution of the non-canonical (non-SMAD) TGFβ pathway in the AV calcification requires further investigation.

Overall, our findings suggest that klotho is an important mediator of TGFβ signaling *in vivo* in adult aortic valves (Figure 7). Both TGFβ1 and TGFβ-dependent canonical (pSMAD2/3) and non-canonical (p38 MAPK, ERK1/2 MAPK) pathways are activated in the absence of klotho. This suggests that klotho restrains TGFβ ligand-receptor-dependent TGFβ signaling and that increased TGFβ-induced activation of SMAD2/3 and/or SMAD1/5 somehow drives the AV calcification in klotho-deficient mice. This is consistent with the observation that partial genetic inactivation of TGFβ1 and *SMAD3* (this study), and BMPR1A (56) can block the development and progression of AV calcification in klotho-deficient animals. Our observations are further supported by *in vitro* evidence that klotho can bind to the TGFβ Type II receptor complex, thereby contributing to the inhibition of TGFβ1 signaling (22, 77). In conclusion, our results demonstrated that inhibition of TGFβ signaling by selectively targeting TGFβ1 or SMAD3 could inhibit AV calcification. This information will be useful in designing safer treatments for CAVD.

## Data Availability Statement

The raw data supporting the conclusions of this article will be made available by the authors, without undue reservation.

## Ethics Statement

The animal study was reviewed and approved by the IACUC University of South Carolina.

## Author Contributions

MA: conceptualization and supervision. MC, AB, MG, IC, ZA, and JJ: methodology. MC, AB, IC, and MA: formal analysis. MC and MA: writing—original draft preparation. IC and MA writing—review and editing. MA and NV: funding acquisition. All authors have read and agreed to the submitted version of the manuscript.

## Conflict of Interest

The authors declare that the research was conducted in the absence of any commercial or financial relationships that could be construed as a potential conflict of interest.

## Publisher’s Note

All claims expressed in this article are solely those of the authors and do not necessarily represent those of their affiliated organizations, or those of the publisher, the editors and the reviewers. Any product that may be evaluated in this article, or claim that may be made by its manufacturer, is not guaranteed or endorsed by the publisher.
